# From across the Seas

**Published:** 1917-03-24

**Authors:** 


### FROM ACROSS THE SEAS.

### Serums and Vaccines.

THE Federal Government having resolved that the serums and vaccines required by Australia shall be manufactured within her borders—ultimately at Canberra, the Federal capital—immediately engaged an English doctor to look after the institute or factory. Dr. Penfold arrived at the end of November, before there was any place where he could work. But the trustees of the Walter and Eliza Hall Fund have come to the rescue and offered the temporary use of such laboratories at the Melbourne Hospital as are not at present in use by the Institute of Research in Pathology and Medicine, founded last year by the Hall Fund. Dr. Cumpston, director of quarantine, and Dr. Penfold are consulting as to the site and construction of the serums factory, and work is to commence shortly; the estimated cost is £12,000, with equipment. It is thought that this factory will supply all the serums and vaccines required by Australia, and thus make her independent of importation.

### A Hospital May Have Too Much Money.

THIS is the moral to be learned from an article in the *Modern Hospital*, St. Louis, by Dr. J. W. Fowler, dealing with a report on economies at the Louisville City Hospital found necessary in the attempt to cope with increased expenditure on the basis of a stationary income. "Nothing so conduces to extravagance," says the editor, "as a knowledge that there is plenty of money. This truism holds good in every walk of life—it is merely one of the weaknesses of human nature. In connection with hospital work, one is sometimes tempted to wonder if the purchase of commodities in large quantities for the sake of lower prices is always wise, because, when everyone knows that 'there is plenty on hand,' waste is almost sure to follow." Broadly speaking, the method advocated by the report is to make a certain quantity of goods and service answer the required purpose, and not to carry out hospital management on the lines that an inexhaustible supply is expected and will be forthcoming.

### The Jeanes Hospital Fund.

THE Jeanes Fund of more than $3,000,000, bequeathed for the establishment of a hospital for cancer and nervous diseases by Anna F. Jeanes, will be used to establish a new and independent general hospital somewhere on the main line within fifteen miles of Philadelphia. This announcement has been made by the chairman of the committee in charge of the selection of a site. Tentative plans for the institution have already been drawn, and several sites are under consideration by the committee. These sites will be inspected in a few weeks and a choice made. Half a dozen big hospitals and medical colleges in Philadelphia and other cities have been bidding for the fund since the death of Miss Jeanes in 1908. Dr. Winford H. Smith, superintendent of the Johns Hopkins Hospital of Baltimore, was selected by the trustees to consider the claims of the various hospitals, and it was finally decided that only by establishing a new and independent hospital could the purposes of the will be carried out.

### Tuberculosis.

THE Federal Committee on Causes of Death and Invalidity in Australia in December presented to the Federal Parliament a long and serious report on tuberculosis in the Commonwealth. During 1914 there were 3,574 deaths from all forms of tuberculosis, or 72.6 per 100,000 of population. There are great discrepancies in percentages in different States and different parts of the same State, and further scientific investigation into these is urged. The committee believes that the disease is much more prevalent than it need be. The committee urges that the sources of the milk supply should be thoroughly investigated, and all methods of its control brought into operation immediately. The necessity for isolation is also strongly urged; it is considered essential that notification should be regular, systematic, and rigidly enforced, and a law is suggested requiring that when a case of tuberculosis is notified every member of the patient's family should be examined and the results reported to the authorities. It is pointed out that the receipt of an invalid pension enables the patient to remain at home, to become a permanent source of infection, and it is considered that legal power should be given to remove such cases to hospitals. The chief recommendation is that Commonwealth and States should co-operate in a vigorous campaign against tuberculosis, commencing, say, at Bendigo, in order to discover the best means of controlling the disease throughout Australia. Bendigo is selected because of the ill eminence it has long held as regards this disease, there named "miner's phthisis." In 1910 the then Mayor of Bendigo and some of the leading citizens started a movement for the establishment of a sanatorium; the sum of £1,500 was collected, a site of over 80 acres was reserved and fenced in, and there it ended; but the money was banked and is earning interest.

### Indian Civil Surgeons and Military Duty.

OF 3,398 Civil sub-assistant surgeons in the various provinces of India, 459 were deputed to military duty up to December 1, 1916. The number dismissed for refusing to accept duty was fifty-four. There were none under this heading in the Punjab Province, which showed the highest percentage of surgeons deputed—viz., 24.85. Of the total number deputed from Bengal, one died of disease in Mesopotamia, and four have reverted to Civil employment on account of ill-health.

